# Author Correction: Origin of outer tropical cyclone rainbands

**DOI:** 10.1038/s41467-023-43547-x

**Published:** 2023-11-21

**Authors:** Cheng-Ku Yu, Che-Yu Lin, Chi-Hang Pun

**Affiliations:** Department of Atmospheric Sciences, National Taiwan University, Taipei, Taiwan

**Keywords:** Atmospheric dynamics, Natural hazards

Correction to: *Nature Communications* 10.1038/s41467-023-42896-x, published online 03 November 2023

The Acknowledgements section was accidentally omitted from this article and should have read:

**Acknowledgements**

The ground-based Doppler radar data used in this study were provided by the Taiwan Central Weather Bureau (Central Weather Administration since 15 September 2023). The TC information, including center locations, RMW and other storm characteristics, was provided by the Joint Typhoon Warning Center. This study was supported by the National Science and Technology Council of Taiwan under Research Grants MOST 111-2111-M-002-017 and NSTC 112-2111-M-002-017.

The original article has been corrected.

