# Supplementary material for: Origin of outer tropical cyclone rainbands
Source: Nat Commun. 2023 Nov 3;14:7061. doi: 10.1038/s41467-023-42896-x (PMC10624872; doi:10.1038/s41467-023-42896-x)
Supplement: Supplementary file 2 — Description of Additional Supplementary Files [file 41467_2023_42896_MOESM2_ESM.pdf]

Description of Additional Supplementary Files

File Name: Supplementary Data 1
Description: A complete list of all outer tropical cyclone rainband (TCR) cases identified and analyzed in this study. Typhoon names and formative date and time for each of the outer TCR cases are also indicated.
